# The 3D Spatial Autocorrelation of the Branching Fractal Vasculature

**DOI:** 10.3390/acoustics1020020

**Published:** 2019-04-09

**Authors:** Kevin J. Parker, Jonathan J. Carroll-Nellenback, Ronald W. Wood

**Affiliations:** 1 Department of Electrical and Computer Engineering, University of Rochester, Rochester, NY 14627, USA; 2 Department of Physics and Astronomy, University of Rochester, Rochester, NY 14627, USA; 3 Department of Obstetrics and Gynecology, University of Rochester Medical Center, Rochester, NY 14642, USA

**Keywords:** scattering, backscatter, ultrasound, tissue, medical ultrasound, fractal vasculature, tissue characterization

## Abstract

The fractal branching vasculature within soft tissues and the mathematical properties of the branching system influence a wide range of important phenomena from blood velocity to ultrasound backscatter. Among the mathematical descriptors of branching networks, the spatial autocorrelation function plays an important role in statistical measures of the tissue and of wave propagation through the tissue. However, there are open questions about analytic models of the 3D autocorrelation function for the branching vasculature and few experimental validations for soft vascularized tissue. To address this, high resolution computed tomography scans of a highly vascularized placenta perfused with radiopaque contrast through the umbilical artery were examined. The spatial autocorrelation function was found to be consistent with a power law, which then, in theory, predicts the specific power law behavior of other related functions, including the backscatter of ultrasound.

## 1. Introduction

In optics, electromagnetics, and acoustics, the behavior of waves within a weakly inhomogeneous medium can be related to properties of the spatial correlation function, a statistical measure of the spatial patterns or fluctuations within the material [1,2]. Both the forward propagating wave and the waves scattered from the inhomogeneities have distributions that have been linked to the spatial patterns of the inhomogeneities. For example, under the Born approximation and assumptions of stationarity, a key integral formula relates the ensemble average measures of backscattered intensity to the material’s spatial correlation function as a 3D Fourier transform operation. A specific case emerges if we consider the parenchyma within organs such as the liver, prostate, or placenta to be the reference media, while the long, cylindrical fluid-filled, fractal branching vessels serve as the weak scattering sites.

Under that hypothesis the fractal branching structure of the fluid-filled vasculature will play a major role in determining the propagation of waves in highly vascularized tissues. However, there are few reported experimental results in the literature that can answer the key question: What is the 3D spatial autocorrelation function of a fractal branching vasculature network from normal soft tissue organs? The question has high significance because the 3D spatial autocorrelation function of these scattering sites drives the statistics of wave propagation, including the millions of ultrasound scans each year derived from backscattered echoes. We address this question directly by applying radiopaque contrast to the vasculature system of a freshly delivered, intact placenta, obtaining high resolution computed tomography (CT) scans, thresholding the 3D data set, and determining local autocorrelation functions within interior regions. In theory, these results provide the quantitative link between the properties of the fractal branching vasculature and physical measures such as scattering and fluctuations in the forward propagating waves.

### Fractal Structures and Scattering

Since the popular work by Mandelbrot [3], the idea of self-similar structures occurring across a wide range of scales has been applied to natural structures. The concept of fractals in biological systems has seen numerous applications [4,5]. The nature of scattering from fractal structures has also received attention, with the concept of a fractal dimension *d_f_* driving power law relations and scattering behavior. For 3D space filling structures, the measured *d_f_* would be less than 3. Lin et al. [6] modeled fractal aggregates and essentially argued that the long wavelength (low frequency or Rayleigh scattering) limit would have the standard *k*^4^ dependence of backscatter where the wavenumber *k* = *ω*/*c*, whereas the short wavelength (high frequency) limit would approach *k*^(4−*d_f_*)^. Javanaud [7] simply invoked a power law relation for scattering using *d_f_* as the scaling power.

Shapiro [8] argued from a number of examples that a fractal structure would have a spectrum (over some range of scales) that follows a power law, and derived a scattering formula for different regimes under that assumption. One conclusion was that over some scale the backscattered intensity would be proportional to *k*^(4+*ν*)^, where *ν* is a constant related to the fractal power law behavior. Sheppard and Connolly [9] considered the optical scattering from random surfaces. Shear wave scattering and loss were attributed to fractal structures in Lambert et al. [10] and Posnansky et al. [11]. The compatibility of these works requires further study.

To summarize, the literature contains a number of theoretical functions in random media that are related to fractals and are linked to different power law relations. However, we are left with some open questions as to the particular autocorrelation function found in practice, and its implications for wave propagation. These are addressed in the following sections.

## 2. Theory of Scattering Applied to Fractal Branching Networks

### 2.1. General Theory

When considering scattering from random media, it can be shown [12–14] that the differential scattering cross section per unit volume *σ_d_*(*k*) and the spatial correlation $b(\hat{r})$ function of the inhomogeneities are related by:

(1) $$\sigma_{d}(k)=k^{4}A\int \int \int b(\hat{r})e^{j2\hat{k}\cdot \hat{r}}\mathrm{dVol},$$

where *k* is the wavenumber, $\hat{r}$ is the vector separation between two points within the ensemble average, and *A* is a constant. Assuming the correlation function is isotropic and simply dependent on the separation distance *r*, the volume integral reduces to:

(2) $$\mathrm{VI}(k)=\frac{2\pi }{k}\int_{0}^{\infty }r\cdot b(r)\sin(2\mathrm{kr})\mathrm{dr},$$

similar to the integral found in Equations (4), (9) and (11) from Parker [15], covering both acoustic and electromagnetic scattering under the Born approximation.

By comparing this to the form of the 3D Fourier transform in spherical coordinates with spherical symmetry J{}3DS [16,17], and shown in Appendix A, we may write:

(3) σd(k)=A⋅k4⋅J{b(r),k}3DS,

where J{b(r),k}3DS=(2π∕k)∫0∞r⋅b(r)⋅sin(2kr)dr. Thus, the calculation of backscattering reduces to the question of the 3D Fourier transform of the isotropic and spherically symmetric ensemble-averaged correlation function of *r*. This spatial correlation function of cylindrical fluid-filled vessels is considered next.

### 2.2. The Long Cylindrical Shape

We considered fractal branching networks of the fluid-filled vasculature and other parallel fluid channels to be comprised of long cylindrical sections, branching out in successive generations from largest to finest.

Assuming an isotropic spatial and angular distribution of each generation of fractal branching structures, the goal was to average the autocorrelation function of a basic element across all angles of incidence with respect to the propagating wave and across all size scales from very small micro-channels of fluid to the largest arteries and veins that can exist within the organ. Specifically, we will examine the fluid-filled cylinder of radius *a*:

(4) f(r)={κ0r≤a0r>a}F(ρ)=κ0⋅a⋅J1[2πa⋅ρ]ρ

where *κ*_0_ is the fractional variation in density plus incompressibility, assumed to be ≪ 1 consistent with the Born formulation, *F*(*ρ*) represents the Hankel transform, which is the two-dimensional Fourier transform of a radially symmetric function, and *ρ* is the spatial frequency.

First, assuming the fluid-filled cylinder is long in the *z*-axis, then the shape is one-dimensional and its autocorrelation can be obtained from the inverse Hankel transform of the square of the shape’s Hankel transform:

(5) $$B_{\mathrm{cyl}}(r)=2\pi \int_{0}^{\infty }\rho \cdot F{\left(\rho \right)}^{2}J_{0}(2\pi r\cdot \rho )d\rho ,$$

where *ρ* is the spatial frequency. Second, we assumed that cylinders of this kind exist within a fractal geometry in an isotropic pattern. Therefore, copies of this were interrogated by the forward wave over all possible angles. Thus, we formed a 3D isotropic correlation function for the shape by conversion to spherical coordinates.

**Figure 1.**
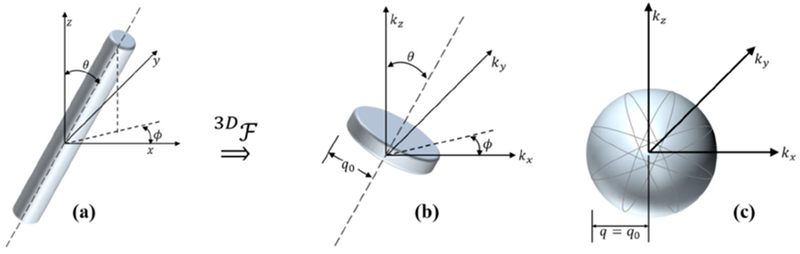
A cylindrical function (**a**) and its Hankel transform represented in 3D Fourier transform space (**b**). Rotations around spherical coordinates similarly rotates the corresponding transform, leading to a spherically symmetric ensemble average (**c**).

Consider first, one infinitely long cylinder with a material property *f*(*r*)—symmetric—as shown in Figure 1a and tilted at some arbitrary angle in a spherical coordinate system. It has a 3D transform that is a thin disk (delta function *k_z_* but shown with finite thickness to make the graphic easier to draw and visualize). Its transform is symmetric and given by the Hankel transform of order zero F(ρ)=H{f(r),ρ}. A particular radius of value of *q*_0_ is shown for reference.

Now, if we have an ensemble average of *F*^2^(*ρ*) across many cylinders at random angles (orientations) as shown in Figure 1a,b, but consider them as independent and uncorrelated (a cloud of sparsely spaced scattering cylinders), then the ensemble average 3D transform is formed from the disk function adding up over all realized angles. In the limit, any cylindrical radius *q*_0_ in Figure 1b forms a spherical shell in the ensemble average (Figure 1c).

**Figure 2.**
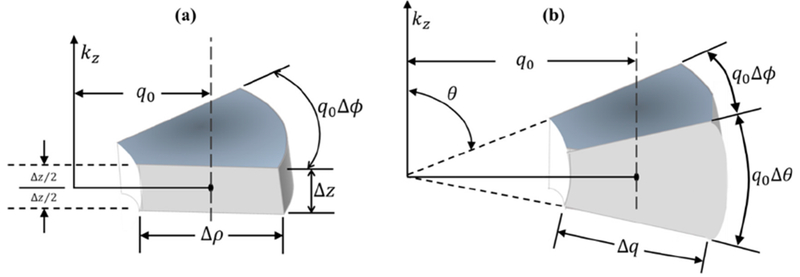
Differential volume element around point *q*_0_ in 3D curvilinear coordinates; cylindrical coordinates in (**a**), and spherical coordinates in (**b**).

Thus, the ensemble average simply takes the Hankel transform function *F*^2^(*ρ*) found in Figure 1b and populates the spherically symmetric 3D transform *B_s_*(*q*), shown in Figure 1c. However, there is a scaling of the cylindrical transform function over the spherical ensemble average. Specifically, the 3D spatial Fourier transform of the long cylinder oriented along the *z*-axis can be expressed as:

(6) F2(kx,ky,kz)=J{b(r)}3DS=δ(kz)H0{b(r),ρ}=δ(kz)F2(ρ)

where $\rho^{2}=k_{x}^{2}+k_{y}^{2},H_{0}\{\}$ is the Hankel transform of order zero [16], and *δ*(·) is the Dirac delta function. Next, the thin disk (delta function) in cylindrical coordinates represented as *F*^2^(*ρ*_0_)*δ*(*k_z_*) must be converted to a thin disk in spherical coordinates *B_s_*(*q*_0_)*δ*(*θ* − *π*/2) as considered in Figure 2, where the common radius *ρ*_0_ = *q*_0_ and Δ*ρ* = Δ*q*. Of importance is the relative scaling of the functions within the conversion to spherical coordinates.

In the limit of a small element size, the integral of each function, or Riemann middle sum, around a point *q*_0_ on a plane orthogonal to the *k_z_* axis will be:

(7) $$q_{0}\cdot F^{2}(q_{0})\Delta \rho \Delta \phi \;\text{for Figure 2a}\;q_{0}^{2}\cdot B_{s}(q_{0})\Delta q\Delta \phi \;\text{for Figure 2b},$$

where the sifting property of the Dirac delta function in curvilinear coordinates is used in the Δ*z* and Δ*θ* directions, respectively. In spherical coordinates, this result is independent of *θ* (see Equation (17) of Baddour [17]). By setting these equal and thus independent of the coordinate system, and setting Δ*ρ* = Δ*q*, we find:

(8) $$B_{s}(q)=\frac{F^{2}(q)}{q}.$$

Thus, with reference to Equation (3), we conclude that:

(9) σd(k)=A⋅k4⋅J{b(r),k}3DS=A⋅k4(F2(k)k)=Ak3F2(k),

where $F^{2}(k)={\left(H_{0}\{f(r),k\}\right)}^{2}$.

Finally, the cross-correlation of an ensemble of these elements and all other (larger and smaller) elements within a fractal structure needs to be derived. The simplest assumption that can be made is that each scattering element has an autocorrelation function with itself that has been determined (above), and that within the ensemble average the cross terms with all other branches (larger and smaller within the fractal structure) are simply a small constant that is nearly invariant with the position and therefore can be neglected except for spatial frequencies nearing zero. Under that very simplistic assumption, the overall autocorrelation function is given by the sum (or integral in the continuous limit) of the different sizes’ correlation functions over all generations of branches, weighted by their relative numbers (number density in the continuous limit). Fractal structures in 3D and volume filling in 3D may be characterized by number density functions *N*(*a*) that are represented by *N*_0_/*a^b^*, where *a* is the characteristic radius of the canonical element, *N*_0_ is a global constant, and *b* is the power law coefficient [18]. Within this framework, the overall correlation function is:

(10) $$B_{T}(r_{s})=\int_{0}^{\infty }N(a)B_{\mathrm{sph}}(r_{s})\mathrm{da}=\int_{0}^{\infty }\frac{N_{0}}{a^{b}}B_{\mathrm{sph}}(r_{s})\mathrm{da}$$

where *N*(*a*) is the number density of elements as a function of size *a*. Alternatively, in the 3D transform domain:

(11) $$B_{\mathrm{TR}}(k)=\int_{0}^{\infty }N(a)\left[F_{r}^{2}(k,a)∕k\right]\mathrm{da}.$$

For the specific case of the fluid cylinder, this becomes:

(12) $$B_{\mathrm{TR}}(k)=\frac{1}{k}\int_{0}^{\infty }\left(\frac{N_{0}}{a^{b}}\right){\left(\frac{\kappa_{0}{\mathrm{aJ}}_{1}[2\pi \mathrm{ak}]}{k}\right)}^{2}\mathrm{da}=f_{1}(b)k^{\left(b-6\right)},$$

where *f*_1_(*b*) is a function of *b*, and with reference to Equation (9), we find that the predicted backscatter is:

(13) $$\sigma_{d}(k)=A\cdot k^{4}\left(f_{1}(b)k^{\left(b-6\right)}\right)\;=A\cdot f_{1}(b)\cdot k^{\left(b-2\right)}.$$

The autocorrelation function *B*(Δ*r*) is also found from the inverse Fourier transform of Equation (12) to be *B*(Δ*r*) = *C* · *f*(*b*)/*r*^*γ*^ and where *γ* = *b* − 3 for the convergence of the inverse transform integral, 5 > *b* > 3, and where *C* is a constant. Note that the integral operators (transform, average over angles, and average over radii) are all linear so the order of these operations can be transposed for computational ease.

**Figure 3.**
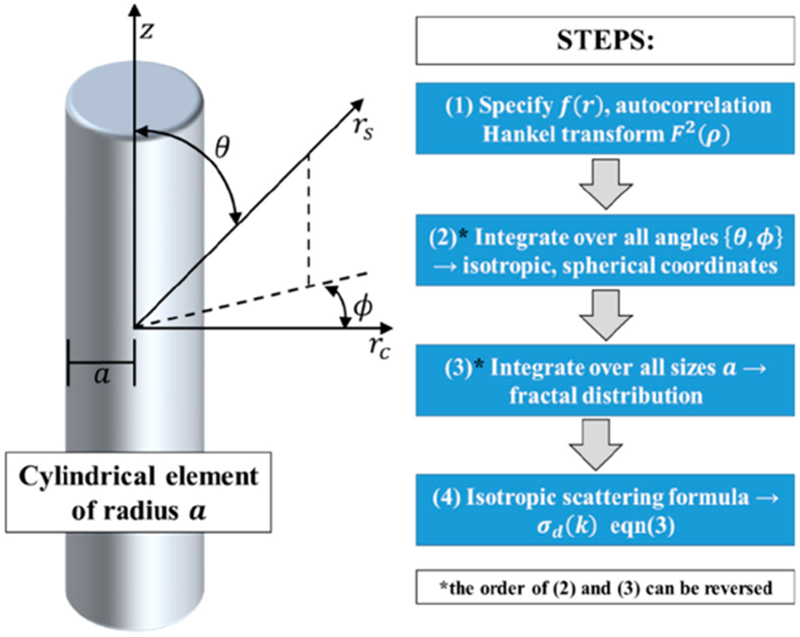
Derivation of isotropic scattering from a cylindrical element.

Our general approach is shown in Figure 3.

## 3. Methods

The fetal side placental perfusion was adapted from dual lobular placental perfusion methods previously published [19–21]. Briefly, human placentae from normal term deliveries were obtained within 5–10 min of delivery and examined for tears and gross lesions. Within 25 min, the umbilical artery and vein were cannulated near the insertion of the cord on the surface of the chorionic plate with five French umbilical catheters for perfusion at 10–15 mL min^−1^ with a finger pump. Flow rate was adjusted to maintain fetal vessel pressure at ~60 mmHg. The fetal perfusate consisted of M199 media without phenol red (Gibco) modified by the addition of dextran (35–45 kDa; 30 mg/mL fetal), D-Glucose (2 mg/mL), sulfamethoxazole (80 μg/mL), trimethoprim (16 μg/mL) gentamicin (52 μg/mL), and heparin (20 USP IU/mL). The fetal perfusate was gassed with 20% O_2_/75% N_2_/5% CO_2_ in a 250 mL vessel and bubbles trapped before delivery to the placenta. The cannulated placenta was placed in a plastic bag and immersed in a 37 °C water bath for an hour followed by Doppler and ultrasound elastography experiments as described in McAleavey, et al. [22]. At the conclusion of these experiments, the placenta was perfused with a 37 °C suspension of 30% barium sulfate in 1% agarose prepared from a 60% emulsion oral contrast suspension (Barium-Liquid E-Z-Pague; Bracco) diluted with a 2% agarose in water solution with a gelling temperature of 35 °C. Perfusion continued until no further change was apparent. The placenta was then immersed in 10% neutral buffered formalin for fixation before imaging with a Philips Brilliance 64 computerized axial tomography system. The slice dimension was 768 × 768 pixels, each 0.25 × 0.25 mm; slice thickness: 0.67 mm, spacing between slices: 0.33 mm.

## 4. Results

**Figure 4.**
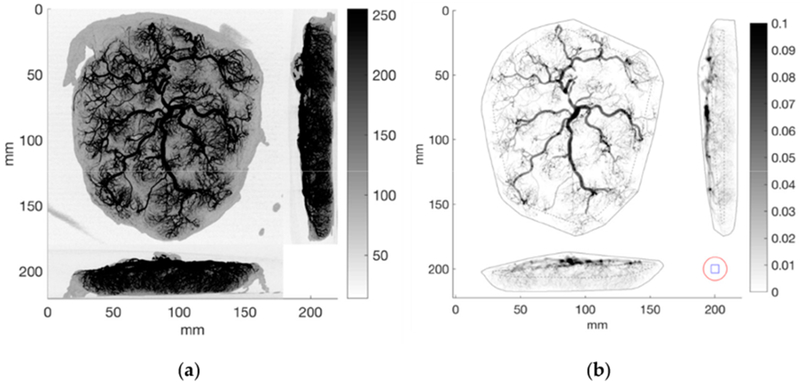
(**a**) Maximum intensity projections of the raw data along the three primary axes, (**b**) Projections of the binary thresholded data along the three primary axes. Shown also is the convex hull (solid black line) and sampling region (dashed black line). The blue square in the bottom right shows the 6 mm × 6 mm autocorrelation window and the red circle 9 mm in radius shows the region used to calculate the spectrum for each sample point.

The raw data set (shown in the maximum intensity projections in the left panel of Figure 4) was thresholded using a constant threshold limit of 180. The threshold was set at the minimum level necessary to zero out the poorly vascularized edges of the placenta, while maintaining well connected branches. Projections of the thresholded binary data can be viewed in the right panel of Figure 4. Also shown in the right panel of Figure 4 is the convex hull of the placenta (solid black line) as well as the boundary of the sampling region (dashed black line). The sampling region contains locations whereby small autocorrelation sample cubes (6 mm on a side, or 27 × 27 × 21 voxels) can be translated along any axis or diagonal by 6 mm without being outside the whole placenta’s boundary. For reference, the size of the autocorrelation cubes is shown in the bottom right corner of the right panel of Figure 4, and the surrounding region that contributes to the autocorrelation spectrum is shown by the red circle of radius 9 mm.

From within the sampling region, 1000 locations were chosen randomly as the center coordinate of an autocorrelation window. Shifts were applied over all possible translations up to 6 mm, and the autocorrelation function *B*(Δ*r*) = 〈*I*(*x*, *y*, *z*) * *I*(*x* + Δ*x*, *y* + Δ*y*, *z* + Δ*z*)〉 was calculated, where *I*(*x*, *y*, *z*) is the thresholded computed tomography (CT) intensity, as a binary function {1,0}, and $\Delta r=\sqrt{\Delta x^{2}+\Delta y^{2}+\Delta z^{2}}$. The normalized autocorrelation *B*(Δ*r*)/*B*(0) was then calculated and used in subsequent analyses.

**Figure 5.**
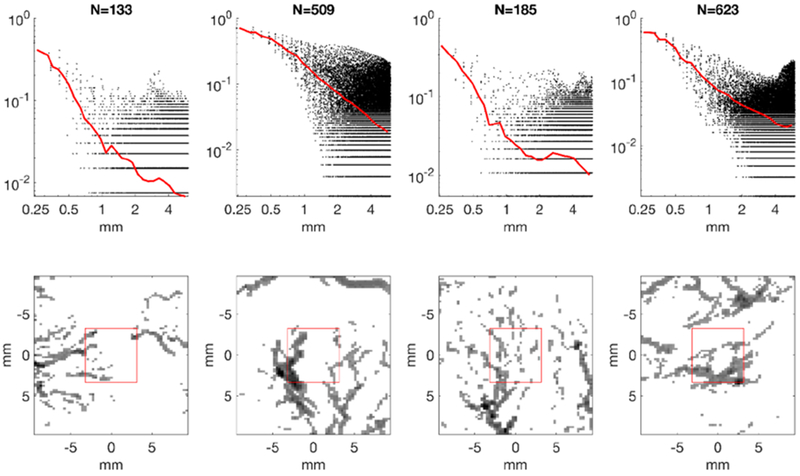
The bottom row shows 4 of the 1000 regions and the surrounding neighborhood. The top row shows, for each region, the normalized autocorrelation for each displacement (dots) as well as the average for each radial bin for a range of *N* = *B*(0) (red line).

Individual locations’ autocorrelation functions were found to be dependent on the number of samples included within the 6 mm local cube, and this is related to the structure(s) included. For example, a box centered on a large arterial segment has many pixels above threshold, and has a rather slow decorrelation since the object *I*(*x*) is large. At the other extreme, if a box is centered on a region with no obvious vascular architecture and only a few small isolated points above threshold, the decorrelation is steep as a function of Δ*r*. These extremes point to the inherent difficulties in characterizing a multi-scale process with limited sample volumes and limited resolution. For many sample locations, however, a branching structure was included, and this corresponded to numbers of voxels *N* above threshold between approximately *N* = 100 and 1000 (out of 15,309 voxels per cube). These characteristic types are shown in Figure 5.

**Figure 6.**
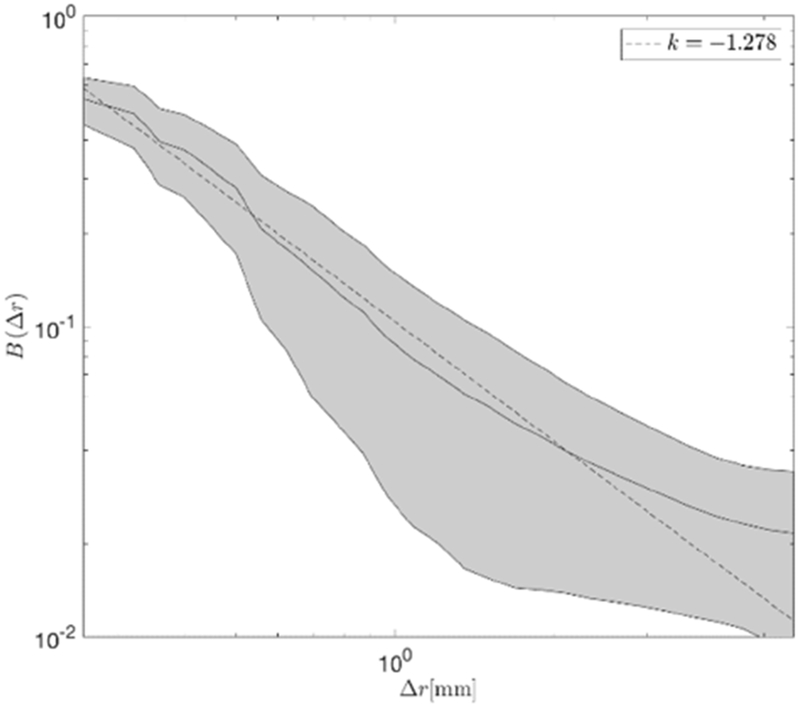
Average normalized autocorrelation function for regions with 100 < *N* < 1000. The grey area is bounded by ±1 standard deviation across all sample locations included in this set.

There is also a theoretical question relating to the nature of the autocorrelation function: Can it reasonably be described as a power law function? To address this, power law functions were fit for autocorrelation lags Δ*r* from 0 to 6 mm. The results of the power law coefficient for *B*(Δ*r*) = *B*_0_ · (Δ*r*)^*γ*^ are plotted in Figure 6. The minimum mean squared error fit to a power law provides an exponent of approximately −1.3. Some indication of a flattening at larger separation distances, greater than 4 mm, was seen however larger multiscale analyses would be required to determine if that was a general result indicating a tendency of the branching vasculature correlation function at longer ranges.

We find some variation in the power law fits for the complete range of lags that was dependent on the lower and upper bounds on voxel counts chosen, as shown in Table 1. The bounds were varied to exclude cases that were extreme in either containing too few voxels above thresholds or too large of a structure to be meaningful, however the final average did depend on the particular values chosen, but clustering around *γ* = −1.3 for a reasonable range of populated autocorrelation boxes.

## 5. Discussion

### 5.1. Consequence of the Model and Power Law

For soft tissue scattering, a number of studies have characterized the optical and acoustical measurements of tissue specimens. Specifically, most carefully calibrated ultrasound backscatter results from soft tissues report increasing backscatter with frequency. Generally this can be fit to a power law with an exponent of 1 < *γ* < 2. For example, Campbell and Waag found liver backscatter to increase as *f*^1.4^ over the medical imaging band of 3–7 MHz [23] consistent with other pioneering reports from the early 1980s [24–28]. Other examples of increasing backscatter vs. frequency in livers are demonstrated in [29] extending up to 25 MHz, and in Lu et al. [30], also in livers. Similarly, for other tissues, including sarcoma and carcinoma models, the increase in backscatter vs. frequency was reported in Oelze and Zachary [31] and for thyroids in Rouyer et al. [32]. Thus, in assessing our theoretical model, Equation (3), we are seeking a prediction of a backscatter power law fit of an exponent slightly larger than one, given a relatively isotropic fractal fluid network scattering power law of *b* − 2. In that case, it is clear that the power law *b* governing the number density of vessels must be greater than 3. This is plausible, although reported data has to be considered in light of the methodologies used; this was considered in Section 5.2. We also note for completeness that for *F*[*q*]^2^/*q* in spherical radial frequency being proportional to *q*^(*b*−6)^, also corresponds in the spatial domain to an autocorrelation function proportional to $1∕r_{s}^{b-3}$, where 3 < *b* < 5. For our placenta measurements, *γ* = −1.3 = 3 − *b* implies *b* = 4.3, also implying backscatter would increase as *k*^2.3^ from Equation (13), over some frequency range.

Although our resolution was limited by the CT system and contrast enhancement, optical studies can obtain finer resolution. Schmitt and Kumar [33] studied phase contrast images of mouse liver histology slides and found a power law power spectrum behavior for spatial frequencies down to at least 1/10 microns.

Similarly, Rouyer et al. [32] studied thyroid tissue using broadband ultrasound systems and by replotting their data we can estimate a power law behavior between 6 MHz and 15 MHz, corresponding to wavelengths down to 100 microns.

### 5.2. Refinement of Theory vs. Experiments

A more exact comparison of theory to experiment will require greater precision on two fronts, the backscatter vs. frequency power law, and the fractal branching power law of the fluid channels, for specific organs. For example, with respect to liver, Campbell and Waag [23] reported a backscatter proportional to the frequency to the 1.4 power over a range around 5 MHz for beef liver samples. These measurements and the analytical treatment of all system effects were highly rigorous, yet confirmation of these results over a larger bandwidth and in conjunction with imaging of the samples (ensuring avoidance of any unwanted ligaments or trapped bubbles, for example) would help to refine this estimate.

The fractal branching behavior assumed in Equation (10) is a continuous number density of cylinders represented as proportional to *N*_0_/*a^b^*; this forms a major assumption of this framework, yet is not known precisely. In reviewing the literature results, it must be kept in mind that there are major distinctions running through different analyses, principally 2D vs. 3D, and also the type of measurement utilized. Fractal dimension is limited by topographical dimension, so 2D analysis of slices or projections will have a lower dimension than corresponding 3D measurements. Full 3D measurements at high resolution are rare and difficult due to the demands on resolution and sheer size of the imaging data. Some high quality and high resolution 3D assessments of the branching vasculature have been rigorously acquired for the brain [34] and the placenta [35], however to our knowledge there have not been any published results from 3D fractal analyses of the liver vasculature at high resolution.

Adding to the uncertainty about the important power law parameter *b*, which defines the number density of cylindrical vessels as a function of their radius, over the ensemble and in linear dimensions, is the wide variety of measurement styles that are typically reported. This is a major source of possible misinterpretation. Our power law parameter *b*, which governs the number density of vessels as a function of diameter, is not the same as the fractal dimension measured by sandbox or related box counting techniques. Even in the cases of older literature reports where the number of vessels were painstakingly counted (see Figure 1 of [18] indicating a power law of approximately −2.7 over some older studies, Table 1 of [36] indicating a power law of approximately −1.3 in pulmonary veins, or Table 4 of [37] suggesting a power law of −2.5 in pulmonary arteries), there are important methodological details that can swing the assessment. For example, many counting schemes derive the number of vessel vs. branch generation number 1, 2, 3 … as ordinal numbers. The slope of this curve is not the same as our number density vs. radius curve. Other schemes count “greater than or equal to” a varying radius, which corresponds to an integration. Since the integration of 1/*a^b^* is proportional to 1/*a*^(*b*−1)^, this scheme inherently shifts and reduces the power law parameter by one. Similarly, a scheme of “binning” together vessels by proportional limits over a log scale will shift the power law. For example, counting all the vessels within ±15% of 0.1 mm, then all those within 15% of 1 mm, then all those within 15% of 10 mm, will effectively integrate the distribution within these bins, again converting the continuous distribution to a discrete set with 1/*a*^(*b*−1)^ relative distribution. For all these reasons, it is plausible that some literature assessments of branching vascular parameters, using different methodologies, may not be the same as number densities defined in our manner by the parameter *b*. Clearly, high resolution data sets in 3D are required to better define this important factor in soft tissues such as liver, thyroid, prostate, and others. The particular 3D shapes and autocorrelation functions from these different organs may differ from the results found in the placenta, since the 3D placenta has a unique relationship with the maternal uterine blood flow, so organ-specific parameters are needed to form baseline values.

## 6. Conclusions

High resolution contrast CT studies of a normal placenta’s branching vasculature in 3D demonstrated a power law behavior for the 3D spatial autocorrelation function with an exponent close to −1.3. Correspondingly, a theoretical hypothesis about scattering from tissue was generated from a primitive cylindrical shape representing the plausible distribution of extracellular fluids and blood in long fluid-filled channels throughout normal soft tissue. Assuming a wide range of diameters of the cylindrical fluid spaces and a macroscopically isotropic distribution over some region of interest within the organ, the predicted backscatter is of the form of a power law with dimension greater than one. This matches some observations about the nature of vessels and measurements of backscatter from soft tissues and is consistent with the power law autocorrelation function measured experimentally from the placenta.

Pathologies that affect tissue morphology would be expected to modify this model in significant ways, but it is prudent to first establish an accurate working model of normal soft tissue scattering. More precise measurements of the key parameters will be required to fully test this hypothesis and prior models based on spheres.

## Figures and Tables

**Table 1.** Average normalized autocorrelation power law fits as a function of upper and lower bounds on occupied voxels, excluding extremely empty or filled samples.

Lower Bound *N*	Upper Bound *N*	Average Power Law *γ*
10	1000	−1.6
10	10,000	−1.5
100	1000	−1.3
100	10,000	−1.3
100	100,000	−1.3

## Appendix. Particular Form of Fourier Transform Pairs

Consistent with Bracewell [16], we use the following convention:

(a) Fourier transform in one dimension:
  (A1) $$F(s)=\int_{-\infty }^{\infty }f(x)e^{-i2\pi \mathrm{xs}}\mathrm{dx}=J\{f(x)\}\;f(x)=\int_{-\infty }^{\infty }F(s)e^{+i2\pi \mathrm{xs}}\mathrm{ds}$$
(b) Fourier transform/Hankel transform for cylindrical symmetry:
  (A2) $$F(\rho )=2\pi \int_{0}^{\infty }f(r)J_{0}(2\pi \rho r)\mathrm{rdr}=H\{f(r)\}\;f(r)=2\pi \int_{0}^{\infty }F(\rho )J_{0}(2\pi \rho r)\rho d\rho$$
(c) Three-dimensional Fourier transform with spherical symmetry:
  (A3) F(q)=2πa∫0∞f(r)sin(2qr)rdr=J{f(r)}3DSf(r)=2πr∫0∞F(q)sin(2qr)qdq

Within this convention, the transform variable has units of cycles/distance. Furthermore, the 3D spherical transform is identical to the kernel for Born scattering, Equations (1)–(3). Finally, using these definitions, we find that a 3D spherically symmetric power law function *b*(*r*) = 1/*r^b^* has the transform:

(A4) $$B(q)=2^{b-1}\cdot \pi \cdot \Gamma [2-b]\sin\left[\frac{b\pi }{2}\right]q^{\left(b-3\right)}\text{for1}<b<3,$$

where Γ is the gamma function. Thus, for example, if *b* = 3/2, an eigenfunction of the transform occurs: J{1r3∕2}3DS=cq3∕2.
